# Role of PTPα in the Destruction of Periodontal Connective Tissues

**DOI:** 10.1371/journal.pone.0070659

**Published:** 2013-08-05

**Authors:** Dhaarmini Rajshankar, Corneliu Sima, Qin Wang, Stephanie R. Goldberg, Mwayi Kazembe, Yongqiang Wang, Michael Glogauer, Gregory P. Downey, Christopher A. McCulloch

**Affiliations:** 1 Matrix Dynamics Group, Faculty of Dentistry, University of Toronto, Toronto, Ontario, Canada; 2 Division of Pulmonary and Critical Care Medicine, Department of Medicine, National Jewish Health and the Integrated Department of Immunology, University of Colorado, Denver, Colorado, United States of America; Centro de Investigacion y de Estudios Avanzados del Instituto Politecnico Nacional, Mexico

## Abstract

IL-1β contributes to connective tissue destruction in part by up-regulating stromelysin-1 (MMP-3), which in fibroblasts is a focal adhesion-dependent process. Protein tyrosine phosphatase-α (PTPα) is enriched in and regulates the formation of focal adhesions, but the role of PTPα in connective tissue destruction is not defined. We first examined destruction of periodontal connective tissues in adult PTPα^+/+^ and PTPα^−/−^ mice subjected to ligature-induced periodontitis, which increases the levels of multiple cytokines, including IL-1β. Three weeks after ligation, maxillae were processed for morphometry, micro-computed tomography and histomorphometry. Compared with unligated controls, there was ∼1.5–3 times greater bone loss as well as 3-fold reduction of the thickness of the gingival lamina propria and 20-fold reduction of the amount of collagen fibers in WT than PTPα^−/−^ mice. Immunohistochemical staining of periodontal tissue showed elevated expression of MMP-3 at ligated sites. Second, to examine mechanisms by which PTPα may regulate matrix degradation, human MMP arrays were used to screen conditioned media from human gingival fibroblasts treated with vehicle, IL-1β or TNFα. Although MMP-3 was upregulated by both cytokines, only IL-1β stimulated ERK activation in human gingival fibroblasts plated on fibronectin. TIRF microscopy and immunoblotting analyses of cells depleted of PTPα activity with the use of various mutated constructs or with siRNA or PTPα^KO^ and matched wild type fibroblasts were plated on fibronectin to enable focal adhesion formation and stimulated with IL-1β. These data showed that the catalytic and adaptor functions of PTPα were required for IL-1β-induced focal adhesion formation, ERK activation and MMP-3 release. We conclude that inflammation-induced connective tissue degradation involving fibroblasts requires functionally active PTPα and in part is mediated by IL-1β signaling through focal adhesions.

## Introduction

Tyrosine phosphorylation of proteins is an important, post-translational regulatory mechanism in cell signaling that regulates many critical processes including cell proliferation, differentiation, migration and adhesion to the extracellular matrix [1], [2]. The extent of tyrosine phosphorylation is determined by a balance between the activities of protein tyrosine kinases and protein tyrosine phosphatases (PTPs), molecules which either phosphorylate or dephosphorylate tyrosine residues, respectively. PTPα is a trans-membrane, receptor-like PTP with two intracellular catalytic domains [3] that is involved in cell transformation and differentiation as a result of its ability to activate Src-family kinases [4], [5]. PTPα has been implicated in regulating integrin-induced auto-phosphorylation of the focal adhesion kinase [6] and the activation of Rac-1 leading to increased turnover of focal adhesions [7]. The phosphatase activity of PTPα in particular is required for IL-1β signaling that leads to Ca^2+^ release from the endoplasmic reticulum [8] and ERK activation, both of which are required for MMP-3 release from fibroblasts [9].

Periodontal diseases are high prevalence infections of humans and other mammals that are associated with inflammation and irreversible destruction of periodontal connective tissues. IL-1β, one of the pro-inflammatory cytokines that has been implicated in mediating periodontal destruction, is particularly abundant in inflamed periodontium [10] and may be important for host defense against periodontal pathogens [11]. Increased concentrations of IL-1β are associated with enhanced levels of IL-6 and TNFα and other pro-inflammatory factors such as reactive oxygen species and PGE_2_, as well as increased expression of MMPs [12] that enable extracellular matrix degradation and tissue remodeling. Alternatively, IL-1β can also activate NF-κB-dependent anti-apoptotic genes [13], which may contribute to the maintenance of gingival fibroblast populations. Notably, gingival fibroblasts express very high levels of IL-1 type 1 signaling receptors [14] that are concentrated in the focal adhesions of these cells when cultured [14]. Further, IL-1β treatment up-regulates collagen synthesis in cultured dermal fibroblasts [15] and in chondrocytes [16]. Thus in anchorage-dependent cells such as gingival fibroblasts, IL-1β may help to balance the production and degradation of extracellular matrix proteins, thereby maintaining tissue structure in inflamed periodontium.

Naturally-occurring periodontal destruction can be modeled in mice and other small rodents by introduction of silk ligatures around the necks of molar teeth, a maneuver that promotes microbial biofilm formation and periodontal inflammation and destruction. As MMP-3 is important for remodeling of inflamed gingival tissues [17] and in periodontal tissue destruction [18], we used ligature-induced periodontitis and cultured fibroblasts to examine respectively, the roles of PTPα in periodontal tissue destruction and IL-1β signaling leading to MMP-3 expression.

## Materials and Methods

### Reagents

Bovine plasma fibronectin (FN), poly-L-lysine (PLL), bovine serum albumin (BSA) and REDExtract-N-Amp™ Tissue PCR kit were from Sigma-Aldrich (St. Louis, MO). Nucleic acid gel stain RedSafe™ was purchased from FroggaBio (Toronto, ON). Recombinant human IL-1β and TNF-α, as well as mouse monoclonal anti-MMP-3 antibody were obtained from R&D Systems (Minneapolis, MN). Rabbit polyclonal antibody against MMP-3 and biotinylated anti-rabbit antibody were purchased from Abcam (Cambridge, MA). Antibody microarrays to human matrix metalloproteinases (MMPs) and tissue inhibitors of MMPs were purchased from RayBiotech Inc. (Norcross, GA). The smart pool siRNA targeting PTPα, DharmaFECT Reagent 1 and bicinchoninic acid (BCA) assay kit were purchased from Thermoscientific (Lafayette, CO). AllStars negative control (scrambled) siRNA was from Qiagen (Valencia, CA). The active conformation of β1 integrin level was detected on mouse fibroblasts with a neo-epitope rat monoclonal antibody (clone 9EG7; BD Biosciences; Mississauga, ON). PTPα was assessed with immuno-affinity purified rabbit antibody that recognizes the D2 domain of PTPα (Millipore; Temecula, CA). Total ERK1/2 was measured with a rabbit polyclonal antibody (Cell Signaling; Beverly, MA) and phospho-ERK1/2 was detected with a mouse monoclonal antibody (Cell Signaling; Beverly, MA). Antibody directed against the HA tag was obtained from Bethy Laboratories (Montgomery, TX). Biotinylated anti-rat antibody and Cy2- and Cy3-conjugated streptavidin were obtained from Jackson Immuno Research Laboratories (West Grove, PA). The transfection reagent FuGENE 6 was from Roche Diagnostics (Indianapolis, IN).

### Cell Culture

Human gingival fibroblasts were cultured in minimal essential medium (α-MEM) containing 10% fetal bovine serum. NIH 3T3 cells were grown in Dulbecco’s modified Eagle’s medium (DMEM) containing 10% calf serum. Immortalized mouse embryonic fibroblasts (MEFs) expressing PTPα (PTPα^WT^) or null cells (PTPα^Null^) were provided by Dr. Jan Sap (University of Copenhagen, Denmark; [19]). Cells were cultured in DMEM supplemented with 10% fetal bovine serum. Genetically modified NIH3T3 fibroblasts that express HA-tagged wild-type PTPα (NIH3T3^PTPα^) and Y789F unphosphorylatable mutant PTPα (NIH3T3^Y789F^) under control of a doxycycline sensitive repressor were obtained from Dr. David Shalloway (Department of Molecular Biology and Genetics, Cornell University, Ithaca, NY) and were generated as previously described [20]. These cells were grown in Dulbecco’s modified Eagle medium containing 5% fetal bovine serum in the presence of 5 ng/ml doxycycline. Prior to experiments doxycycline was removed (14–16 hours before) to allow expression of recombinant PTPα. All media used during passaging were also supplemented with 146 units/ml penicillin G, 50 µg/ml gentamycin sulfate and 0.25 µg/ml amphotericin B (Fungizone).

### Genotyping

PTPα wildtype (PTPα^+/+^) and PTPα knockout mice (PTPα^−/−^) provided by Dr. Jan Sap (University of Copenhagen, Denmark; [19]) were bred on a 129SvJ background and were genotyped by digesting the tail clips using REDExtract Tissue PCR kit following manufacturer’s standard PCR procedure. The following primers were used in the identification of the insert: P1∶5′-CCTGACTCTGGAGCCCACC-3′, P2∶5′-CGCATCTCGGGTACCTGC-3′ and P3∶5′-GGAGTTCTTCGCCCACCCC-3′. The PCR products were then separated on a 2% agarose gel containing DNA dye called RedSafe™ (1∶20000 dilution) and visualized using the gel imager FluorChem IS-8900 (Alpha InnoTech; Santa Clara, CA).

### Mouse Experimental Periodontitis

All procedures for inducing experimental periodontitis used in this study were approved by the University of Toronto Animal Care and Use Committee (Animal Care Protocol #9528, Faculty of Medicine, University of Toronto) in agreement with the ethical and legal guidelines set forth by the Ontario’s Animals for Research Act and the federal Canadian Council on Animal Care (CCAC). Accordingly, mice were maintained in a facility with 12 hour light/dark cycle (Division of Comparative Medicine, University of Toronto) and were fed sterile food and distilled water *ad libitum*. Mice were anesthetized with intraperitoneal injections of ketamine (100 mg/kg) and xylazine/rompun (10 mg/kg) ensuring minimal discomfort to the animals. Ligatures (9–0 silk) were tied around the second left maxillary molar (M2) of each animal and carefully introduced into the gingival sulcus to induce experimental periodontitis. The right maxillary second molar was not treated and served as an internal control. Three weeks after ligation animals were euthanized by CO_2_ inhalation and the maxilla were collected for analyses.

### Morphometry

The mouse skulls including maxillae were collected 21 days after the placement of ligatures and were defleshed by a dermestid beetle colony (Dr. Seymour; Royal Ontario Museum; Toronto, ON). Following freeze fumigation, the skulls were stained with 1% methylene blue in water (w/v) and were imaged using PixeLINK® CCD camera attached to a stereomicroscope. The photomicrographs of the buccal and palatal aspects of right (healthy) and left (ligature) maxillary molars were obtained at 6.3×magnification. Horizontal bone loss was measured from the distance between the cemento-enamel junction (CEJ) and the alveolar bone (AB) at mesial, mid and distal sites of both buccal and palatal aspects of each tooth using ImageJ software.

### Micro-computed Tomography (µCT) Analysis

Dried and defleshed mice skulls that were stained with methylene blue above were scanned at a resolution of 11.6µm in all three spatial dimensions using a Skyscan 1172 µCT scanner and analyzed with CT-Analyzer version 1.6.1 software (Toronto Centre for Phenogenomics). The frontal plane of the specimen was set parallel to the X-ray beam axis. Using WCIF-ImageJ software, stacks of reconstructed scans were Gaussian low–pass filtered and the plane connecting the tips of disto–palatal cusps of right and left M2 were positioned horizontally before acquiring the orthogonal view for each specimen. Using the coronal and sagittal views, mesio-buccal, disto-buccal, mesio-palatal and disto-palatal sites of second molars were identified. Alveolar bone loss was assessed by measuring the distances from the CEJ to the alveolar bone crest on the trans-axial view. The measurements were repeated three times per site, and the results are presented as the percent bone loss compared to control (right) side.

### Histomorphometry

Following a 21 day post-ligation period, maxillae designated for routine histology were fixed in 10% formalin, decalcified in hydrocholoric acid, dehydrated in graded alcohols, cleared in xylene and embedded in paraffin. Serial sections (5µm thickness) in the coronal (frontal) plane were stained with hematoxylin and eosin (H&E). These sections were used to determine the mid-position of the second molar. Five sections from this mid-position were selected for each of Gomori’s trichrome and Picrosirius red (PSR) staining. Sections stained with Gomori’s trichrome and PSR were then imaged under bright-field and polarizing light, respectively, with a 20×objective on a Nikon Eclipse TE300 microscope. Using ImageJ, the distances between the CEJ to the alveolar bone crest were measured to estimate alveolar bone loss in Gomori’s trichrome stained sections and the gingival thickness was measured from the apical terminus of the gingival epithelium to the alveolar bone crest on PSR-stained sections. Using the RGB measure Plug-in of Image J, 24-bit color polarized images of the PSR sections were then analyzed in the above region of interest and the amount of red pixels were measured.

### Immunohistochemistry

Paraffin sections were deparaffinized, rehydated and blocked with 10% normal goat serum in PBS containing 1% BSA. Antibodies were diluted in PBS containing 1% BSA and incubations were performed in a humidified chamber. Section were incubated first with rabbit anti MMP-3 (1∶50) overnight at 4°C, followed by incubations at room temperature for one hour each with biotinylated goat anti-rabbit (1∶100) and then with Cy-2 conjugated streptavidin (1∶400). Following mounting with ProLong® Gold antifade reagent containing DAPI (Invitrogen), sections were imaged by fluorescence microscopy using a 40×objective on a Nikon Eclipse TE300 microscope.

### Short Interfering RNA

NIH 3T3 cells were plated at ∼30% confluence 24 hours prior to transfection and were incubated with 10 nM scrambled siRNA or PTPα smart pool siRNA and DharmaFECT Reagent 1 for 48 hours following the manufacturer’s instructions. Cells were trypsinized and re-plated on FN (10 µg/mL) for 3 hours in medium containing 2% serum before stimulating with 40 ng/mL IL-1β or vehicle control in serum-free medium for various time points. Whole cell lysates were collected and protein concentrations were determined by BCA assay. Equal amounts of total proteins from each treatment condition were separated on 10% acrylamide gels and immunoblotted to estimate the effectiveness of PTPα knockdown (KD) and IL-1β-induced ERK activation.

### Immunocytochemistry and TIRF Analysis

NIH 3T3 cells subjected to siRNA treatment for ≥48 hours were trypsinized and re-plated on FN-coated glass-coverslip-attached dishes (MatTek Corporation; Ashland, MA) for 3 hours in the presence of low serum (2%), before treatment with vehicle (PBS) or with IL-1β (40 ng/mL) in serum-free medium for 15 or 30 min. Alternatively, cells that were prepared as described above, with or without PTPα -KD, were allowed to spread in medium containing 2% fetal bovine serum on FN-coated MatTek dishes in the presence or absence of IL-1β (40 ng/mL) for 30 or 60 minutes. At the end of the treatment period, cells were fixed with 4% paraformaldehyde for 10 min, permeabilized with 0.2% Triton-X-100 and blocked for 1 hour in 0.2% BSA. Cells were then incubated at room temperature with rat anti mouse-CD29 (clone 9EG7; 1∶100 dilution) a neo-epitope antibody that recognizes the active conformation of β_1_ integrin on mouse cells followed by biotinylated anti-rat antibody (1∶200 dilution) and lastly with Cy3-conjugated streptavidin (1∶400 dilution) for 1 hour each. Immunostained cells were visualized by total internal reflection fluorescence (TIRF) microscopy (Leica Microsystems) at a Z-axis depth of 90 nm above the coverslip. The mean number and area of focal adhesions per cell were quantified with Leica MetaMorph software (>20 cells per condition) as described previously [21].

### Transient Transfection with PTPα Constructs

PTPα^WT^ MEFs were seeded in 6-well plates at a density of 1×10^5^ cells/well 24 hours prior to transfection in order to obtain 30–40% confluence on the day of transfection. As outlined in the manufacturer’s guidelines, transient transfections were carried out with FuGENE 6 transfection reagent. HA-tagged DNA constructs of WT PTPα, PTPα lacking the D2 domain and PTPα lacking the D1 and D2 domains were kind gifts from Dr. J. den Hertog (Hubrecht Laboratory, Netherlands Institute for Developmental Biology, Utrecht, Netherlands). Briefly, cells were incubated with HA-tagged DNA constructs complexed with Fugene 6 reagent at a ratio of 1∶3, for 5–7 hours at 37°C. Subsequently, PTPα^Null^ MEF cells that were transfected with WT PTPα (referred to as PTPα^Rescue^) or with PTPα lacking the D2 domain (referred to as PTPα^ΔD2^) or with PTPα lacking the D1 and D2 domains (referred to as PTPα^ΔD1&D2^) for approximately 48–72 hours. Cells were then trypsinized, allowed to spread on FN (10 µg/mL) for 3 hours in medium containing 2% serum and then treated with IL-1β (40 ng/mL) or vehicle control. Following treatments, whole cell lysates and/or conditioned media were collected and the protein concentrations were assessed by BCA. Conditioned media were concentrated 20x using Amicon-Centricon (Millipore) filters with 10 kDa molecular weight cut-off. Equal amounts of total proteins from each treatment condition were separated on acrylamide gels and the transfection efficiency (using an HA antibody), and the levels of IL-1β-induced ERK phosphorylation and MMP-3 release were estimated by Western blot.

### Quantitative Real-Time PCR

Following the manufacture’s protocol total RNA was extracted from cells using the RNeasy Mini Kit (Qiagen, Missisauga, ON). Using Nanodrop 1000 (Thermoscientific) the concentration and the integrity of the collected RNA was confirmed prior to PCR analysis. Using iScript™ cDNA Synthesis Kit (Bio-Rad, Hercules, CA) 1 µg of total RNA was reverse transcribed according to the company instructions. Real-Time qPCR was performed on Bio-Rad’s CFX96 Real-Time PCR system using SsoFast™ EvaGreen® Supermix (BioRad, Hercules, CA) with validated mouse primers for GAPDH (Forward 5′-CACACCGACCTTCACCATTTT; Reverse 5′-GAGACAGCC GCATCTTCTTGT) and MMP-3 (Forward 5′-TGGGATTCTGTGAGGATGCCTTA; Reverse 5′-AAAGCCCTTCCCATAGTTGCCAGA). Relative quantification was done using the ΔΔCt method in which the target gene (MMP-3) was normalized to a reference gene (GAPDH) and the fold differences were calculated relative to the non-treatment controls of the NIH3T3^PTPα^ cells (wild type). The data was plotted from the average and S.E.M. values fold changes derived from three independent experiments.

### Isolation of Focal Adhesions

Following previously described procedures [22], cells seeded on poly-L-Lysine coated dishes for 4 hours were incubated with FN (10 µg/mL for 30 minutes at 37°C) coated iron beads (<5 µm diameter) with or without IL-1β (40 ng/mL). Following specified incubation period cells with bound beads were collected with ice-cold cytoskeleton extraction buffer (CKSB: 0.5% Triton X-100, 50 mM NaCl, 300 mM sucrose, 3 mM MgCl_2_, 1 mM Na_3_VO_4_, 1 mM phenymethylsulfonyl fluoride, 1 µM phalloidin and 10 mM PIPES; pH 6.8, along with Sigma Protease inhibitor cocktail at a ratio of 1∶50). Magnetically pelleted beads were washed with ice-cold cytoskeletal extraction buffer three times, re-suspended in 2x SDS-sample buffer and boiled for 5 minutes to dissociate the bead-bound proteins. Equal amounts of bead-associated proteins were immunoblotted for PTPα, vinculin, paxillin, FAK, Src and vimentin.

### Experimental Design and Analysis

All assessments of the mouse periodontitis model were derived from ≥5 specimens. The mean ± S.E.M. were calculated for each measure. Data from *in vitro* experiments are representative of at least three independent repeats using cells from different passages. Numerical data are illustrated on histograms as means ± S.E.M. Student’s *t*-test was performed for comparison of two data sets and for multiple comparisons, analysis of variance was used. By normality tests we determined that the data were Gaussian distributed prior to statistical testing. When appropriate, statistical significance was set at p≤0.05; these p-values are depicted in the table and figures.

## Results

### PTPα Mediates Periodontal Tissue Destruction

PTPα regulates IL-1β signaling in fibroblasts through adhesion-dependent mechanisms that lead to ERK activation and enhanced matrix metalloproteinase-3 (MMP-3) release [23]. As the levels of several inflammatory cytokines, including IL-1β [24] and TNF-α [25] are strongly increased in ligature-induced periodontitis in mice, as well in rats [26], we determined whether PTPα regulates inflammation-mediated destruction of periodontal tissues by placement of silk ligatures around the necks of molar teeth to induce periodontitis [27], [28] using wild type (PTPα^+/+^) or knock out (PTPα^−/−^) mice. First the genotype of mice was confirmed by PCR analysis of tail clips using primers that recognize the insert in the null genotype. Depending on the number and size of PTPα alleles, the genotype was determined from the ∼220 bp band, which represents the WT allele and the ∼350 bp band, which represents the null allele (Figure S1A; left panel). Tissue lysates from lung and gingiva of wild type (PTPα^+/+^) and knock out (PTPα^−/−^) mice were analyzed by Western blot for PTPα which showed absence of PTPα in tissues from null mice (Figure S1A; right panel). Assessments of the morphology of periodontal tissues in untreated mice by histology showed no differences in the distribution of fibroblasts and collagen fibers between the two genotypes (Figure S1B). Likewise no differences were observed in bone mineral density in the presence or absence of PTPα, as determined by µCT scanning (Figure S1C). Assessment of the percent increase of the area of the inflammatory cell infiltrate by image analysis software (WCIF-ImageJ) in ligated tissues compared to the control side showed no significant change between the two mouse strains (Figure S1D).

**Figure 1:**
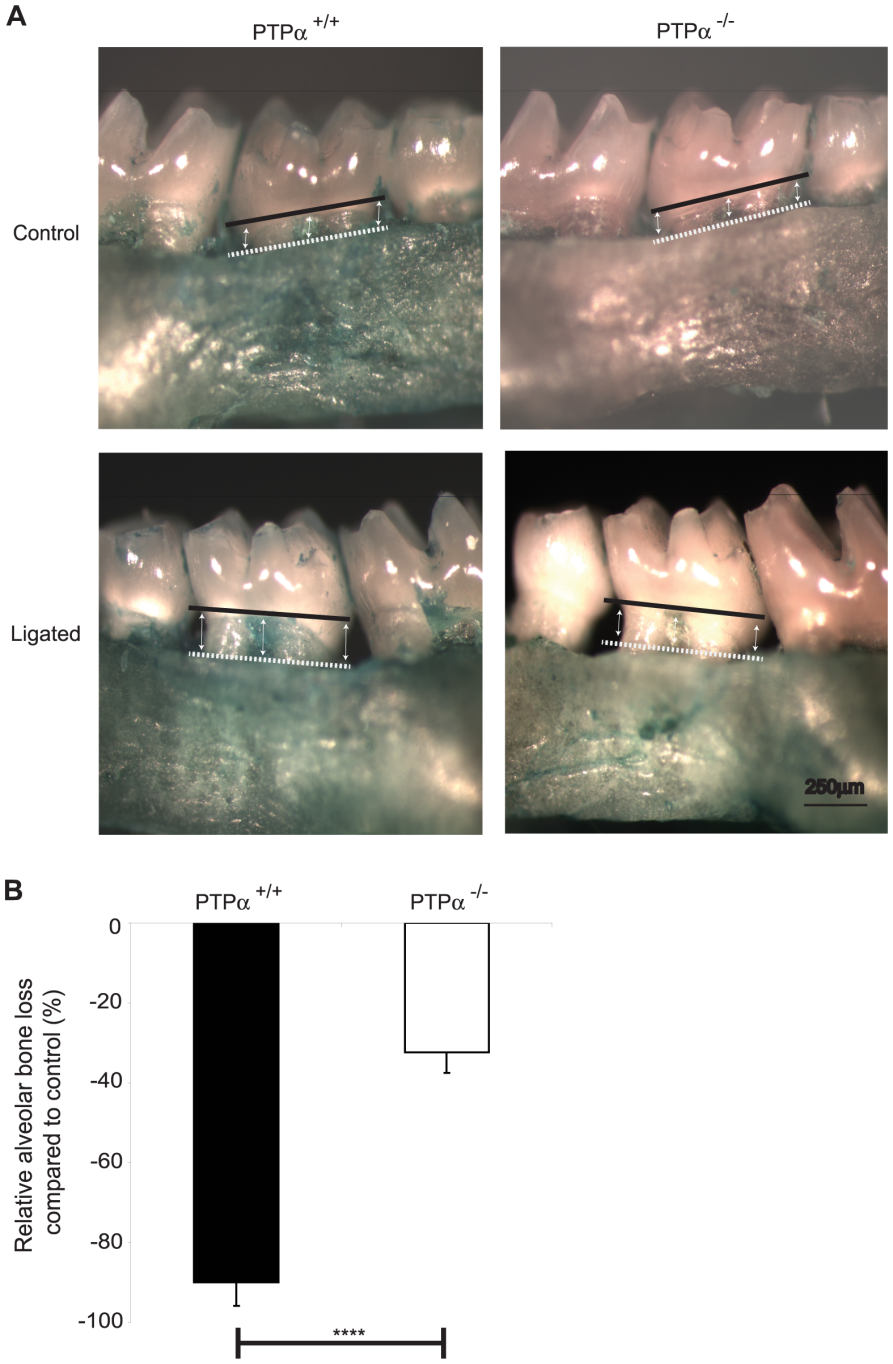
Bone loss analyzed by gross morphometry. (A) Maxilla were imaged with a stereomicroscope. The distance from CEJ (solid black line) to alveolar bone (dashed white line) was measured on ImageJ at mesial, mid and distal sites as indicated on the images, on both palatal and buccal sides. (B) The percent bone loss compared to the non-ligated side was plotted using the mean ± S.E.M. values from 5 maxillae per genotype. ****p<0.0001.

**Figure 2:**
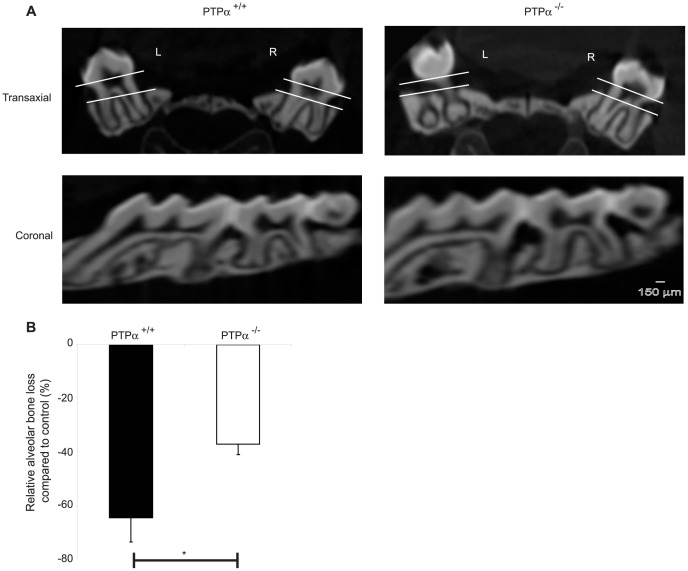
Bone loss analyzed by µCT scanning. (A) Orthogonal views were generated using 3D reconstructed images on WCIF-ImageJ software. Using the coronal view, M2 was chosen and on the transaxial view the distance between CEJ (top line) and alveolar bone (bottom line) was determined on the left (L; ligated) and right (R; control) molars. (B) Relative (%) bone loss compared to the control side was calculated and presented as mean ± S.E.M. data from 5 maxillae per genotype. *p<0.05.

**Figure 3:**
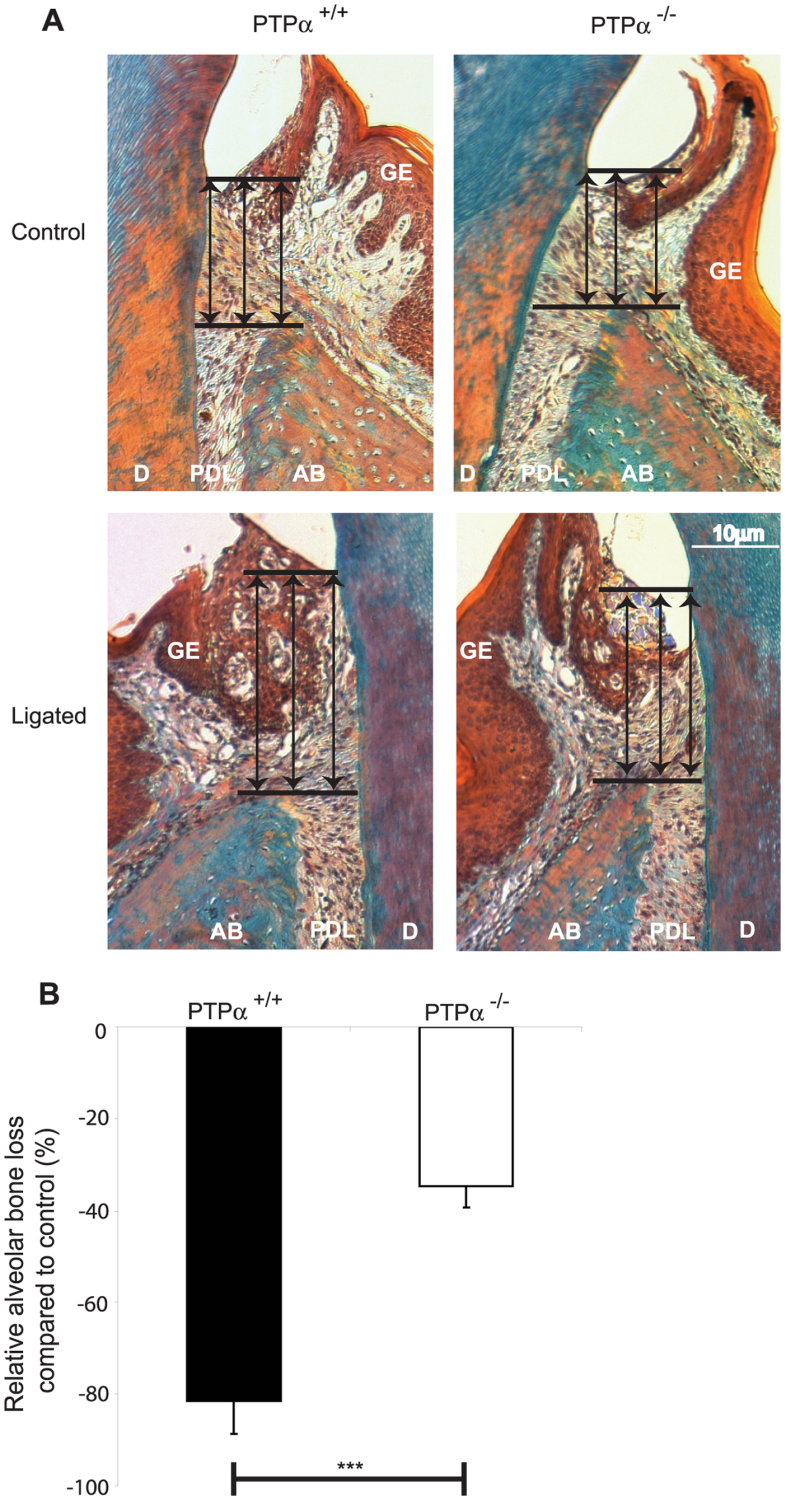
Histomorphometric assessment of bone loss. (A) Gomori’s trichrome stained images were obtained by bright field (20×objective). Tissue designations: AB: alveolar bone, D: dentin, GE: gingival epithelium, PDL: periodontal ligament. (B) The distance from CEJ to alveolar bone was assessed as depicted by the arrows in (A) above, using ImageJ as indicated. The values from 5 samples per strain were analyzed to obtain mean ± S.E.M. of the percent bone loss in relation to non-ligated side. ***p<0.001.

In ligature-treated mice, gross morphometric assessment of methylene blue-stained maxillae revealed marked bone loss on the ligated side compared to the control (non-ligated) side (Figure 1A). The percentage of bone loss on the ligated side compared to the control side was reduced ∼3-fold in PTPα null mice (Figure 1B; p<0.0001). Further, µCT scans of the skulls (Figure 2A) were analyzed by image analysis software as above and relative bone loss (%) was derived from measurements of the distance between the CEJ to the alveolar bone crest. These data indicated that in PTPα null mice, bone loss (%) was reduced by >1.5 fold (Figure 2B; p<0.05). Periodontal tissues were assessed in sections stained with Gomori’s trichrome that differentially stains keratin and cytoplasm as yellow-red while collagen and connective tissue are blue-green (Figure 3A). Bone loss on the ligated side was evident compared to the untreated side in both genotypes (raw data not shown), and, similar to the methylene blue and µCT morphometry data above, there was ∼2 fold less percent bone loss in mice null for PTPα than in WT (Figure 3B; p<0.001).

**Figure 4:**
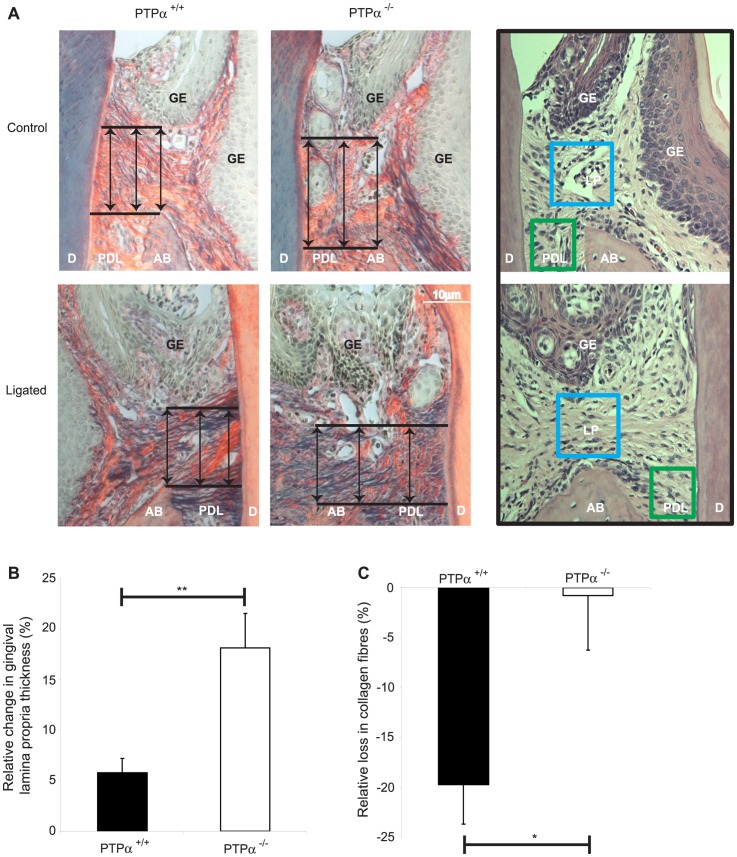
Changes in gingival fiber thickness. (A) Picrosirius-red stained images were obtained using polarized light at 20×magnification. Sampling areas used in 4C and 5B are shown in H&E-stained sections on the right panels. Tissue designations: AB: alveolar bone, D: dentin, GE: gingival epithelium, LP: gingival lamina propria, PDL: periodontal ligament. (B) The gingival lamina propria thickness was measured from the apical extent of gingival epithelium and alveolar bone, as illustrated by the arrows in (A) above, and the percentage change in comparison to control side. Data were plotted with the mean ± S.E.M. values from 5 samples for each strain. (C) Amount of collagen fibers in lamina propria (measured in the area bound by blue box in the inset (A) above) was estimated from RGB levels using Image J and the relative loss compared to control side were plotted with the mean ± S.E.M. values from 5 samples for each strain. *p<0.05, **p<0.01.

**Figure 5:**
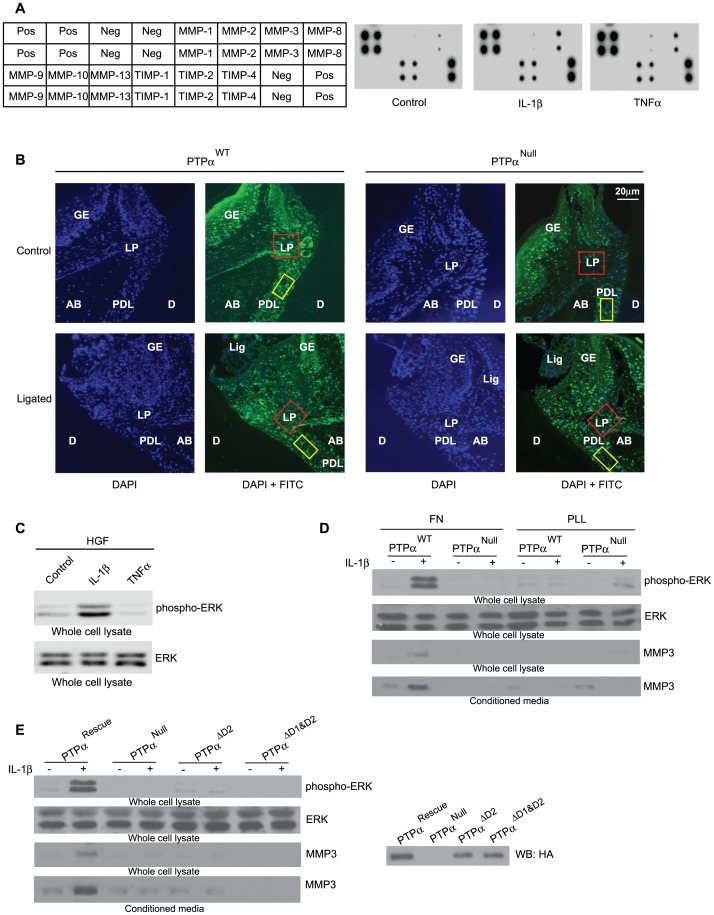
Cytokine signaling in fibroblasts. (A) HGFs grown on fibronectin with 2% serum for 3 hours and then stimulated under serum free conditions with vehicle, IL-1β (40 ng/mL) or TNF-α (10 ng/mL) for 4 hours. Undiluted conditioned media were used to screen for human MMP/TIMP in antibody arrays. Array key is displayed on the left of the blots. (B) Immunohistochemical assessment of MMP-3 expression detected with Cy-2 labeled-streptavidin (green) and the nuclei with DAPI stain (blue) in gingival lamina propria (red box; corresponds to blue box in the inset of Figure 4A) and in periodontal ligament (yellow box; corresponds to green box in the inset of Figure 4A). Tissue designations: Tissue designations: AB: alveolar bone, D: dentin, GE: gingival epithelium, Lig: Ligature, LP: gingival lamina propria, PDL: periodontal ligament. (C) Whole cell lysates from HGFs that were cultured on fibronectin under low (2%) serum and treated in serum free medium with vehicle, IL-1β (40 ng/mL) or TNF-α (10 ng/mL) as in (A) for 15 minutes, were immunoblotted for phospho-ERK and total ERK. (D) Mouse embryonic fibroblasts that are WT (PTPα^WT^) or null (PTPα^Null^) for PTPα were cultured on FN or PLL-coated tissue culture plastic for 3 hours in presence of 2% serum and stimulated with or without IL-1β for 15 minutes. Equal amounts of whole cell lysates, as determined by BCA protein assay, were separated and blotted for phospho-ERK and total ERK. Alternatively, conditioned media collected from cells plated overnight on FN or PLL coated dishes in the presence or absence of IL-1β (40 ng/mL), were concentrated 20x and equal amounts of proteins were loaded onto polyacrylamide gels to detect MMP-3 by Western blot. (E) PTPα^Null^ cells transfected with HA tagged full length construct (PTPα^Rescue^) or the construct lacking D2 domain (PTPα^ΔD2^) or the construct lacing both D1 and D2 domains (PTPα^ΔD1/D2^) as described in the Methods were plated on FN as above and lysates were immunoblotted for phospho-ERK, ERK, MMP-3 and HA tag (right panel inset).

Histological sections stained with picrosirius red stain and viewed by polarized light provided high contrast imaging of collagen fibers (Figure 4A). Assessment of the thickness of the gingival lamina propria (distance from apical gingival epithelium to the alveolar bone crest) as indicated by the arrows in Figure 4A) showed that in wild type mice, the thickness of the gingival lamina propria (including fibroblasts and matrix) was reduced by 3 times compared to PTPα^−/−^ mice (Figure 4B; p<0.01). The H&E-stained sections (Figure 4A; right panels) show the sampling areas in which collagen fibers as illustrated by blue box and MMP-3 as illustrated by blue and green boxes were analyzed, as shown in Figure 4C and 5B, respectively. When the same area was also analyzed in 24-bit color image with the ImageJ Plug-in RGB measure (to quantify the level of the red channel representing amount of collagen fibers), there was >20 times reduction in collagen fibers in the lamina propria of wild type mice compared to null (Figure 4C; *p<0.05).

### PTPα Mediates IL-1β Induced MMP-3 Expression in Fibroblasts

As IL-1β and TNFα are two of the important pro-inflammatory cytokines that contribute to inflammatory connective tissue degradation in periodontitis [29], we determined whether matrix metalloproteinases (MMPs) and tissue inhibitors of MMPs (TIMPs) were stimulated or inhibited by these cytokines. Notably, of the several different cell types found in gingival connective tissues, fibroblasts are particularly important in degradative processes [30] and the release of MMPs. Hence conditioned media from human gingival fibroblasts (HGFs) were screened with human MMP antibody arrays. Cells were allowed to spread on fibronectin for 3 hours in low serum and then treated with PBS (control), IL-1β (40 ng/mL) or TNF-α (10 ng/mL) for 4 hours. These concentrations of cytokines were optimized previously based on study of Ras activation in fibroblasts [31]. IL-1β and TNF-α treatments markedly and selectively increased the release of MMP3 into the medium; the levels of other MMPs and TIMPs were not affected (Figure 5A). In addition, immunohistochemical assessment of tissue sections showed elevated expression of MMP-3 in periodontal tissues subjected to ligation, which was more evident in tissues from PTPα^+/+^ mice than PTPα^−/−^ mice (Figure 5B; areas assessed are outlined by red and yellow boxes in the gingival lamina propria and periodontal ligament, respectively; these sampling areas are also illustrated more clearly in the inset of Figure 4A). Since IL-1β and TNFα signaling involve different signaling pathways, the effect of these cytokines on ERK activation was examined in whole cell lysates from HGFs grown on fibronectin and stimulated with vehicle, IL-1β or TNFα (at concentrations described above for 15 minutes; Figure 5C). Activation of ERK was only seen after stimulation with IL-1β.

IL-1β signaling to ERK in cultured fibroblasts depends on the presence of focal adhesions [32]. Hence the involvement of PTPα in IL-1β-induced signaling was examined using immortalized mouse embryonic fibroblasts that express either PTPα (PTPα^WT^) or that do not (PTPα^Null^). PTPα was required for IL-1β-induced activation of ERK (Figure 5D) but when cells were plated on poly-L-Lysine (PLL), a substrate that prevents the formation of focal adhesions, IL-1β-induced ERK activation was not detectable in cells that expressed PTPα and was barely detected in PTPα null cells, possibly because of non-canonical ERK signaling pathways that are associated with lamellipodia extension [33] (Figure 5D). Similarly, IL-1β enhanced the release of MMP-3 in conditioned media collected from PTPα WT cells, but only when the cells were plated on a matrix (FN) that permitted focal adhesion formation (Figure 5D).

We performed rescue experiments in which PTPα^Null^ cells were transfected with either a full-length PTPα construct (PTPα^Rescue^) or with a PTPα construct lacking the D2 catalytic domain (PTPα^ΔD2^) or with a PTPα construct lacking both D1 and D2 catalytic domains (PTPα^ΔD1&D2^) as described [9]. Although the D1 domain of PTPα harbors almost all of the phosphatase activity [34], [35], [36] and the presence of D1 alone is enough to ensure turnover of focal adhesions [37], we found that it was not sufficient to restore IL-1β-induced ERK phosphorylation and MMP-3 release (Figure 5E; inset on right panel shows the transfection efficacy of the HA tagged constructs described above).

**Figure 6:**
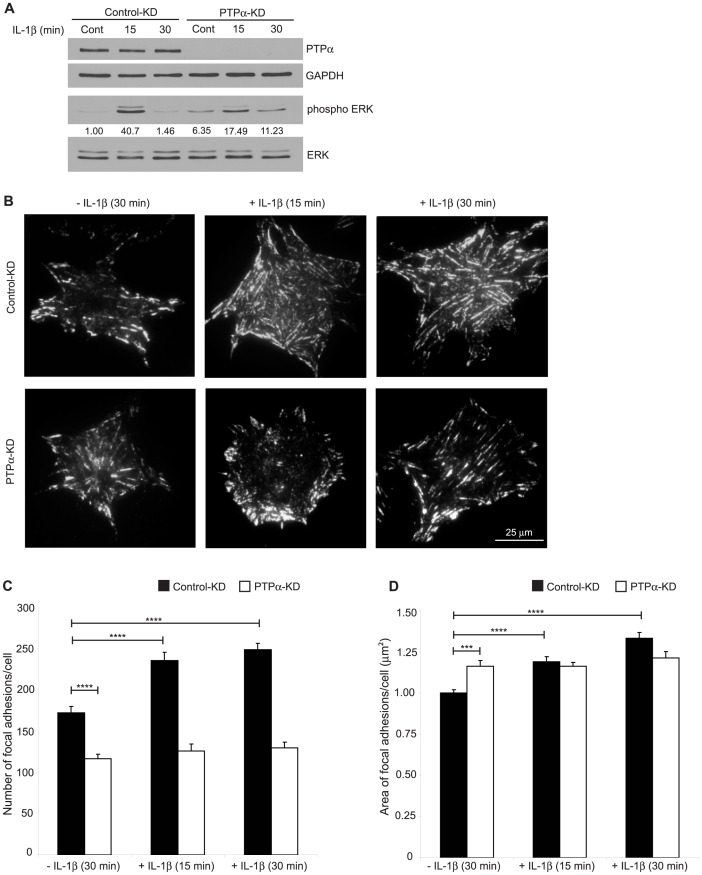
Effect of PTPα on IL-1β-induced ERK activation and sizes of focal adhesions (FA). (A) NIH 3T3 cells transfected with either scrambled or PTPα siRNAs using DharmaFect transfection reagent 1 for 48 hours were trypsinized and re-plated on FN for 3 hours in medium containing 2% serum. After treatment with vehicle (PBS) or with IL-1β (40 ng/mL) in serum-free medium for 15 or 30 min, cell lysates were immunoblotted for PTPα, GAPDH, phospho-ERK and total ERK. Representative blots from three independent experiments were shown along with the densitometry values calculated using ImageJ. (B) Following the same procedure as above cells spread on FN-coated MatTek dishes and were immunostained for active β1 integrins (neo-epitope antibody 9EG7). By TIRF microscopy cells were imaged and analyzed with Metamorph to calculate mean ± S.E.M. of the (C) number of focal adhesions and (D) area of focal adhesions (µm^2^) per cell. ***p<0.001, ****p<0.0001.

The dependence of IL-1β induced ERK activation on PTPα was further confirmed using NIH 3T3 cells that were treated with PTPα siRNA. Following 48 hours of knocked down cells were re-plated on fibronectin and stimulated with IL-1β and whole cell lysates were immunoblotted for phospho-ERK to estimate ERK activation. Densitometry analyses showed that the robust activation of ERK induced by IL-1β was observed in controls compared to cells with knockdown of PTPα (Figure 6A).

We examined the role of PTPα in regulating IL-1β induction of β1-integrin activation in focal adhesions. Focal adhesions were allowed to form and then any additional effect of IL-1 on focal adhesion formation was measured. Control siRNA or PTPα-depleted cells as above were plated on FN-coated dishes for 3 hours and treated with vehicle or IL-1β in serum-free medium, immunostained for activated β1-integrins and analyzed by TIRF microscopy (Figure 6B). In cells with knockdown of PTPα, the number of focal adhesions with activated β1-integrins was reduced (Figure 6C; p<0.0001) but the area of these focal adhesions was increased (Figure 6D; p<0.001). After IL-1β treatment, only cells expressing PTPα displayed increased numbers (Figure 6C; p<0.0001) and areas (Figure 6D; p<0.0001) of focal adhesions compared to controls, indicating that PTPα may recruit specific sets of proteins to focal adhesions in response to IL-1β. In a related experiment, we examined the effect of IL-1 on *de novo* focal adhesion formation. Cells were allowed to spread on FN in the presence or absence of IL-1β for 30 or 60 minutes (Figure S2; Early adhesion). These data showed that IL-1 also enhances IL-1β induction of *de novo* β1-integrin activation in focal adhesions.

**Figure 7:**
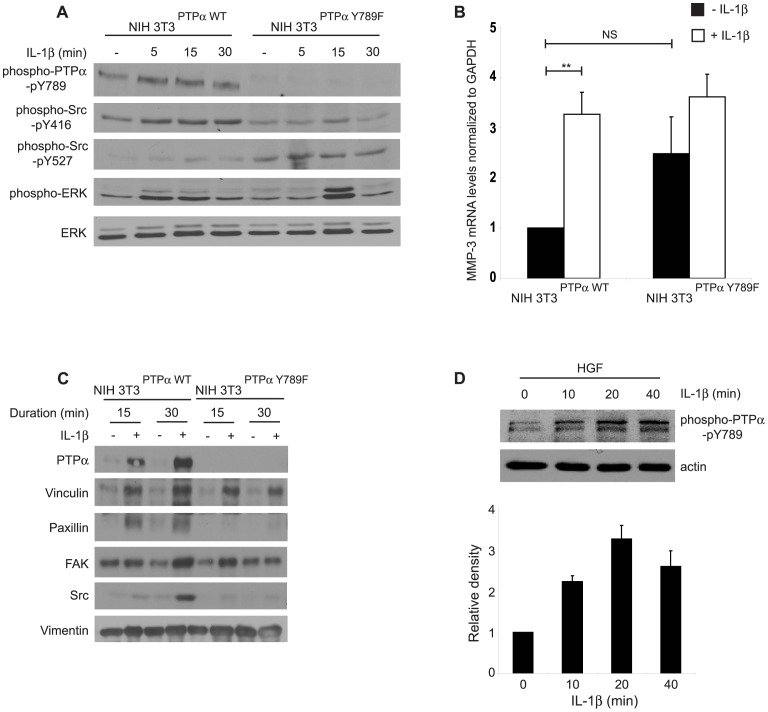
Effect of catalytic and adaptor functions of PTPα in IL-1β signaling. (A) Whole cell lysates collected from NIH3T3 cells genetically modified to over-express wild-type PTPα (NIH3T3^PTPα^) or unphosphorylatable PTPα-Y789F mutant (NIH3T3^Y789F^) were plated on fibronectin and treated with or without IL-1β (40 ng/mL) for the time points indicated were immunoblotted for phospho-PTPα-pY789, phospho-Src-pY416, phospho-Src-pY527, phospho-ERK and ERK. (B) Same cells as above were treated with or without IL-1β (40 ng/mL; 4–5 hours) and analyzed by quantitative RT-PCR as described in Methods. Data are mean±S.E.M. of fold differences relative to the non-treated controls of NIH3T3^PTPα^ in MMP-3 mRNA levels normalized to GAPDH mRNA levels from three independent experiments. (C) The cells described above in panels B and C, were plated on PLL for 4 hours, incubated with FN-coated magnetite beads and treated with vehicle or with IL-1β (40 ng/mL for 15 or 30 minutes). Magnetically-isolated bead-associated proteins were immunoblotted for PTPα, vinculin, paxillin, FAK, Src and vimentin. (D) Whole cell lysates prepared from human gingival fibroblasts grown on FN in the presence or absence of IL-1β (40 ng/mL) for varying time points as indicated, were immuno-blotted for phospho-PTPα-pY789 and actin. **p<0.01, NS: Not significant.

The Tyr 789 residue of PTPα is involved in the regulation of integrin-induced cell spreading and localization of PTPα to focal adhesions [38]. We determined whether a point mutation of this residue would affect the kinetics and extent of IL-1β-induced ERK activation. First, to validate the function of these cells, the activation status of Src was assessed in cells over-expressing wild type (NIH3T3^PTPα^) or non-phosphorylatable PTPα (Y789F). After plating non-phosphorylatable PTPα cells on FN and stimulation with IL-1β, immunoblotting showed that compared with the wild type cells, phosphorylation of the activating Tyr 416 residue of Src was reduced and there was increased phosphorylation of the inhibitory Tyr 527 residue of Src (Figure 7A), indicating reduction of Src activation in the mutant cells because of the loss of the regulatory Y789 residue of PTPα. In response to IL-1β, there was a short-lived increase of ERK phosphorylation (at 15 minutes) in the mutant cells compared to wild type cells in which there was relatively sustained ERK activation (from 5 to 15 minutes; Figure 7A), reflecting the loss of wild type PTPα regulation of integrin function and appropriate localization to focal adhesions of key signaling proteins (see below). We determined whether ERK activation led to MMP-3 release by analyzing mRNA levels of MMP-3. IL-1 induced robust (∼4 times; p<0.01) elevation of MMP-3 mRNA in wild type cells but this increase was not seen in Y789F mutant cells (Figure 7B).

As the Y789 residue is involved in the adaptor function of PTPα via its interactions with Grb-2 [39], we assessed its involvement in IL-1β-induced recruitment of focal adhesion proteins. Analysis of proteins bound to FN-coated beads in Y789 mutant cells showed a reduction of recruitment of certain focal adhesion proteins (paxillin and Src) in response to IL-1β compared with wild type cells (Figure 7C). These results indicate that the phosphorylation of PTPα at Tyr 789 is important for the assembly of focal adhesion proteins that enable appropriate IL-1β signaling leading to ERK activation and MMP-3 expression in mouse fibroblasts. In human gingival fibroblasts we found that IL-1β induces time-dependent phosphorylation of Y789 of PTPα (Figure 7D), indicating that this function-activating residue is indeed involved in IL-1 signaling.

## Discussion

Our main finding is that PTPα plays a central role in the connective tissue destruction of ligature-induced periodontitis possibly through the maintenance of focal adhesion dynamics in fibroblasts that enable IL-1β signaling and MMP-3 expression. The novelty of these findings arises from the employment of the PTPα null mouse for studies of inflammatory destruction of connective tissues, which has not been undertaken previously. Taken together with the bone loss data (∼1.5–3 fold), the destruction of gingival collagen (∼20 fold) in mice expressing PTPα indicates that PTPα is involved in inflammatory processes that drive collagen degradation and alveolar bone loss. Consistent with these findings, infection-induced liver inflammation is associated with increased plasma levels of PTPα but these levels are reduced after treatment, suggesting that PTPα could serve as a diagnostic marker [40] and/or may be required for inflammatory signal transduction leading to connective tissue destruction.

**Figure 8:**
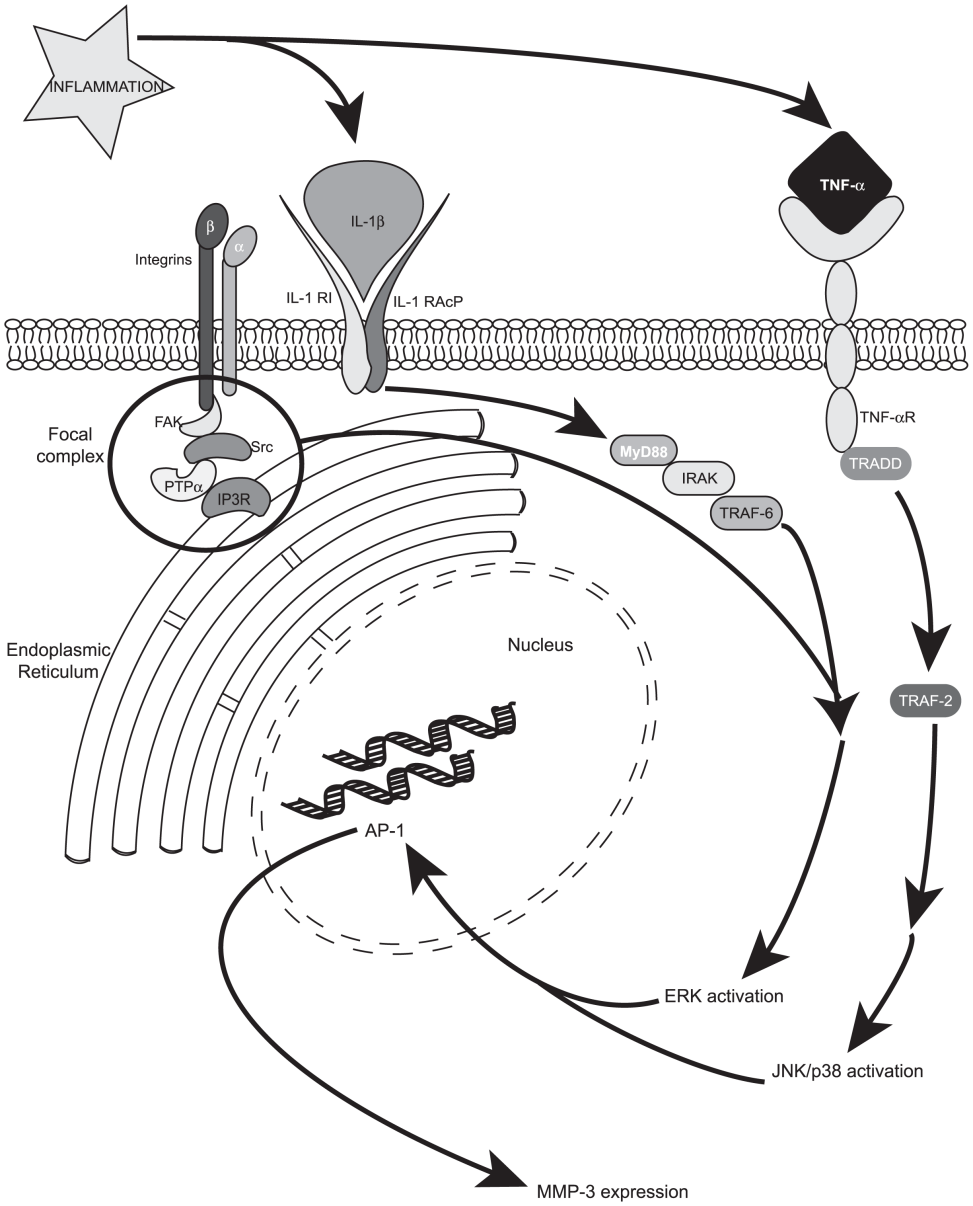
Model of cytokine signaling pathway involved in inflammation. Two of the prominent inflammatory cytokines, IL-1β and TNF-α, signal through MAP kinase (ERK/JNK/p38) activation leading to MMP-3 release via different pathways. Among other focal adhesion proteins, PTPα is required for IL-1β signaling to ERK. Abbreviations used are: IL-1β: interleukin-1β; IL-1R1: interleukin-1 receptor-1; IL-1RAcP: interleukin-1 receptor associated protein; TNF-α: tumor necrosis factor-α; TNF-αR: tumor necrosis factor-α receptor; FAK: focal adhesion kinase; IP3R: inositol 1,4,5-trisphosphate receptor; ERK: extracellular signal-regulated kinase; JNK: c-Jun N-terminal kinase; AP-1: activating protein-1; MMP-3: matrix metalloproteinase-3.

Since the transgenic mice used in this study were global KOs, PTPα was not expressed by many of the cell types in periodontal connective tissues, including fibroblasts and inflammatory cells. Accordingly, the reduction of connective tissue destruction in PTPα null mice could have been mediated by several different cell types. As fibroblasts are the most abundant cell type in gingival connective tissues [41], we focused our *in *vitro studies on fibroblasts including those that expressed or were null for PTPα. Two key inflammatory cytokines, IL-1β and TNF-α, induced MMP-3 expression by gingival fibroblasts; the inflamed mouse tissues in ligated sites also exhibited high levels of MMP-3 immunoreactivity, especially in PTPα^+/+^ mice. However, unlike TNF-α, only IL-1β treatment promoted activation of ERK and only under conditions favorable for focal adhesion formation. The percentage area of the inflammatory cell infiltrate in tissues subjected to ligation compared with controls was similar in PTPα WT and null mice, indicating that PTPα did not exert an influence on recruitment of inflammatory cells to the lesions. Taken together these findings motivated us to focus on the effect of PTPα on IL-1β signaling in fibroblasts. Our data from studies of cultured fibroblasts showed that PTPα is indeed an important mediator of IL-1β signaling, which mediates activation of mitogen-activated protein kinases (e.g. ERK) and MMP-3 expression, which is important for connective tissue destruction in periodontitis [18], [42] (Figure 8).

IL-1β signaling leading to ERK phosphorylation and MMP-3 release in fibroblasts involves PTPα and its association with Src [23] and SHP-2 [9]. In this context, protein tyrosine phosphatases play an important role in inflammatory signaling [43]. In particular, PTPα is critically involved in the regulation of cell adhesion by reinforcing mechanical force-induced integrin cytoskeletal bonds [44] and cell-matrix interactions [4], [6], [45]. Our *in vitro* data showed that in cells expressing PTPα, IL-1β enhances the number and size of focal adhesions with activated β1 integrins. In cells null for PTPα, the focal adhesions were less numerous but were comparable in size to IL-1β-treated cells expressing PTPα, which is in agreement with previous data indicating that absence of PTPα results in formation of super-mature focal adhesions [37], as is seen in cells from Src- [46] or FAK- [47] deficient mice. Notably, the *in vivo* correlate of the focal adhesion, the fibronexus, is found in fibroblasts in inflamed connective tissues [48], [49], suggesting that signaling processes involving focal adhesions may be important for control of inflammation *in vivo.*

We found that deletion of the phosphatase domains of PTPα blocked IL-1β-induced signaling. While the phosphatase activity of PTPα is primarily dependent on the amino terminal catalytic domain, regulatory functions are mediated through the carboxyl terminal domain [36]. Indeed, only cells that expressed intact PTPα were able to respond to IL-1β with enhanced ERK activation and MMP-3 release while the deletion of either the carboxyl catalytic domain or both catalytic domains abolished those signals. Further, we found that perturbation of the catalytic activity of PTPα by point mutations in the D1 (C433S) and D2 (C723S) domains disrupts IL-1β signaling to ERK activation and MMP-3 expression [23], possibly by interfering with the catalytic and/or adaptor function of PTPα [9]. Notably very low levels of ERK phosphorylation in response to IL-1β were seen in cells with PTPα knock down; there was also a transient spike of ERK phosphorylation in NIH3T3^Y789F^ cells. As the cells lacking functional PTPα have impaired spreading, alternative signaling pathways may promote aberrant ERK phosphorylation. Conceivably, the composition of focal adhesions is altered in the absence of functional PTPα, as IL-1β induced recruitment of proteins such as paxillin and Src in particular, to focal adhesion was impaired in the unphosphorylatable PTPα mutant (NIH3T3^Y789F^).

Collectively these data indicate that PTPα is needed for the recruitment of specific proteins to focal adhesions [9], [23], possibly as a result of the catalytic and/or regulatory activities of its domains. These domains in turn may modulate the molecular composition of focal adhesions and as a result, help to differentiate TNFα signaling from the IL-1β signaling processes that are transduced through focal adhesions [50] (Figure 8). Accordingly, focal adhesion formation in cells expressing PTPα is required for IL-1-induced ERK activation and MMP-3 expression. As the notion of exploiting signaling platforms as therapeutic targets is gaining ground [50], PTPα may be a promising target for blocking inflammatory lesions that involve IL-1β signaling.

## Supporting Information

Figure S1 **Establishment of PTPα^+/+^ and PTPα^−/−^ genotype and phenotype.** (A; left panel) Mice tail clips were genotyped as described in Methods section to identify the 350 bp null allele and 220 bp WT allele. (A; right panel) Lung and gingival tissues extracted from PTPα^+/+^ and PTPα^−/−^ mice were homogenized in lysis buffer and following separation on poly acrylamide gels were probed for PTPα and GAPDH. (B) Histological assessment of H&E stained frontal, paraffin sections of non-ligated PTPα^+/+^ and PTPα^−/−^ maxilla. (C) Microcomputed tomography was used to determine the bone mineral density of untreated PTPα^+/+^ and PTPα^−/−^ maxillae. (D) Relative change in percent area of inflammatory cell infiltrate measured in the gingival connective tissue on the ligated side compared to the control side. Data were calculated from measurements obtained using Image J and plotted with the mean percent ± S.E.M. from 5 samples for each strain.
(TIF)

Figure S2 **Effect of PTPα on IL-1β induced nascent adhesion sizes.** (A) NIH 3T3 cells with PTPα or control -KD were re-plated on FN-coated MatTek dishes in presence or absence of IL-1β (40 ng/mL) for 30 or 60 minutes. After immunostaining for activated conformation of β1 integrin (neo-epitope antibody 9EG7), cells were imaged by TIRF microscopy. Metamorph was used to quantify mean ± S.E.M. of the (B) number of focal adhesions and (C) area of focal adhesions (µm^2^) per cell. **p<0.01, ***p<0.001, ****p<0.0001, NS: Not significant.
(TIF)
