# Supplementary figures and images for: Role of PTPα in the Destruction of Periodontal Connective Tissues
Source: PLoS One. 2013 Aug 5;8(8):e70659. doi: 10.1371/journal.pone.0070659 (PMC3734242; doi:10.1371/journal.pone.0070659)

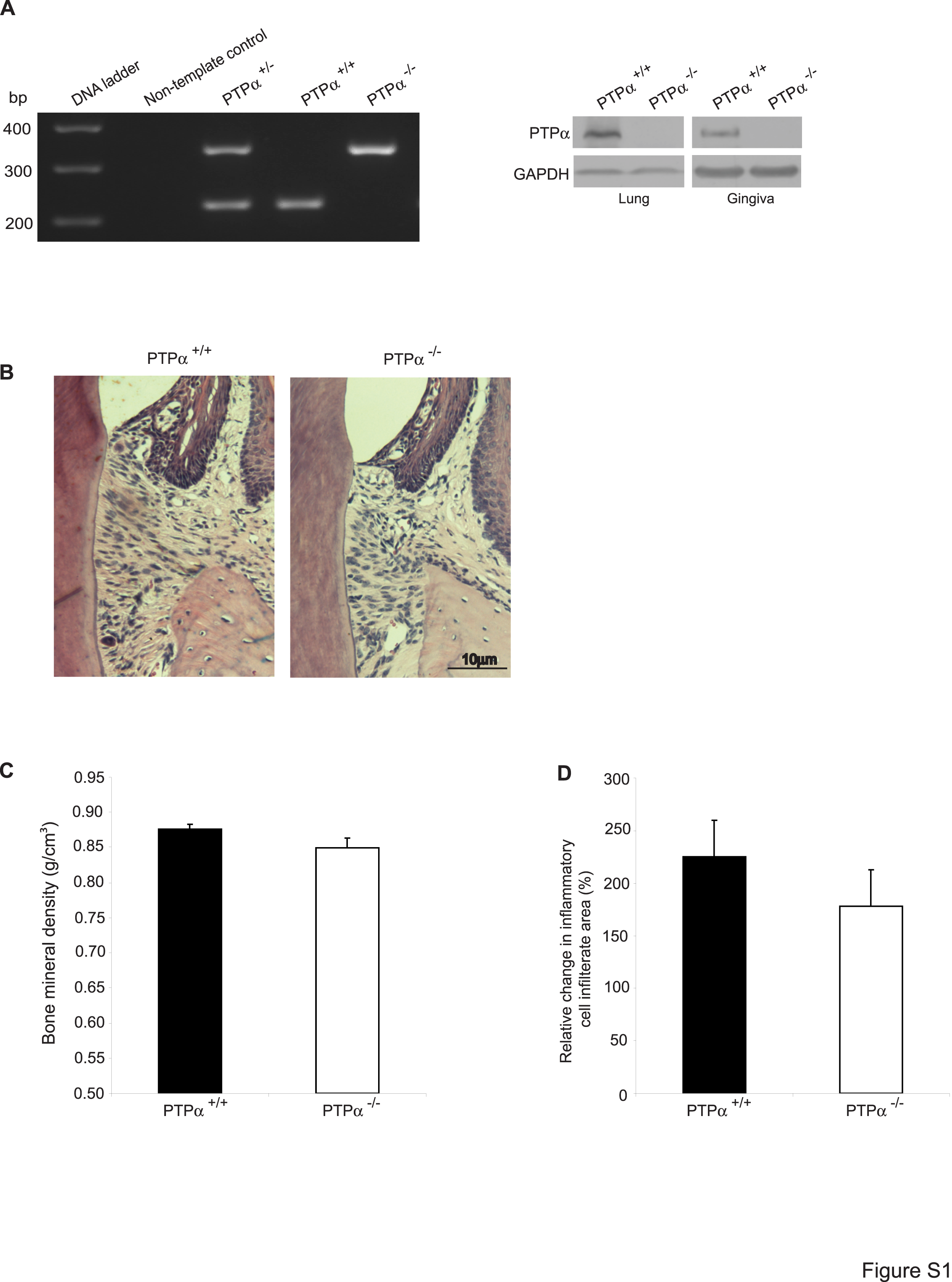

Supplement: Figure S1 — Establishment of PTPα+/+ and PTPα−/− genotype and phenotype. (A; left panel) Mice tail clips were genotyped as described in Methods section to identify the 350 bp null allele and 220 bp WT allele. (A; right panel) Lung and gingival tissues extracted from PTPα+/+ and PTPα−/− mice were homogenized in lysis buffer and following separation on poly acrylamide gels were probed for PTPα and GAPDH. (B) Histological assessment of H&E stained frontal, paraffin sections of non-ligated PTPα+/+ and PTPα−/− maxilla. (C) Microcomputed tomography was used to determine the bone mineral density of untreated PTPα+/+ and PTPα−/− maxillae. (D) Relative change in percent area of inflammatory cell infiltrate measured in the gingival connective tissue on the ligated side compared to the control side. Data were calculated from measurements obtained using Image J and plotted with the mean percent ± S.E.M. from 5 samples for each strain. (TIF) [file pone.0070659.s001.tif]

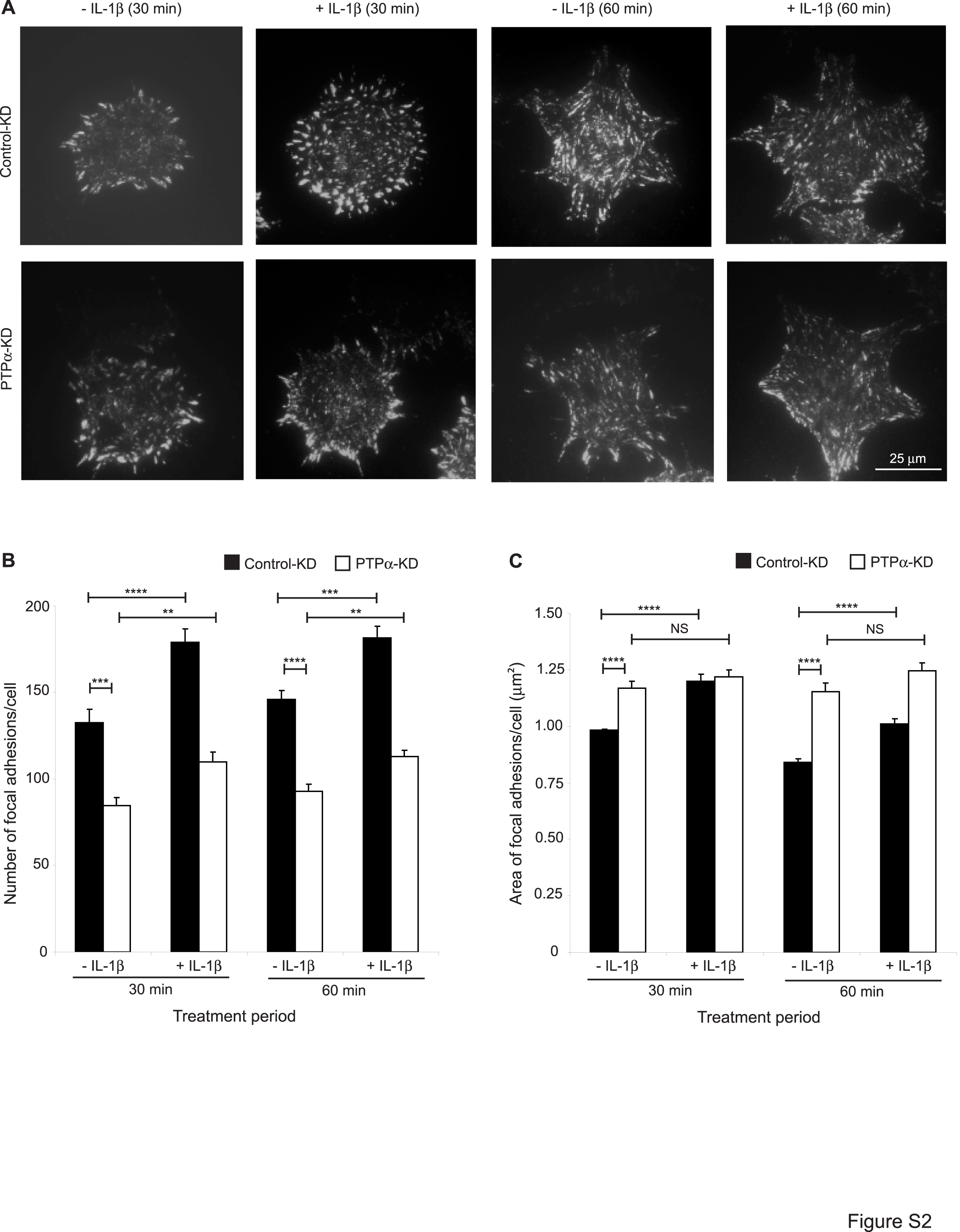

Supplement: Figure S2 — Effect of PTPα on IL-1β induced nascent adhesion sizes. (A) NIH 3T3 cells with PTPα or control -KD were re-plated on FN-coated MatTek dishes in presence or absence of IL-1β (40 ng/mL) for 30 or 60 minutes. After immunostaining for activated conformation of β1 integrin (neo-epitope antibody 9EG7), cells were imaged by TIRF microscopy. Metamorph was used to quantify mean ± S.E.M. of the (B) number of focal adhesions and (C) area of focal adhesions (µm2) per cell. **p<0.01, ***p<0.001, ****p<0.0001, NS: Not significant. (TIF) [file pone.0070659.s002.tif]
